# Urinary sediment mRNA as a potent biomarker of IgA nephropathy

**DOI:** 10.1186/s12882-024-03696-7

**Published:** 2024-11-08

**Authors:** Jin Sug Kim, Geon Woo Kim, Hyeon Seok Hwang, Yang Gyun Kim, Ju-Young Moon, Sang Ho Lee, Junhee Seok, Donghyun Tae, Kyung Hwan Jeong

**Affiliations:** 1 Division of Nephrology, Department of Internal Medicine, Kyung Hee University College of Medicine, Kyung Hee University Medical Center, 26, Kyungheedae-ro, Dongdaemun-gu, Seoul, 02447 Republic of Korea; 2 Division of Nephrology, Department of Internal Medicine, Kyung Hee University College of Medicine, Kyung Hee University Hospital at Gangdong, Seoul, Korea; 3 School of Electrical Engineering, Korea University, Seoul, South Korea

**Keywords:** Biomarker, IgA nephropathy, Urinary mRNA

## Abstract

**Background:**

The quantification of mRNA expression in urinary sediments is a reliable biomarker for various diseases. However, few studies have investigated the clinical relevance of urinary mRNA levels in IgA nephropathy (IgAN). Thus, we investigated the expression of urinary mRNAs and their clinical significance in IgAN.

**Methods:**

Overall, 200 patients with biopsy-proven IgAN, 48 disease controls, and 76 healthy controls were enrolled. We identified the differential expression of mRNAs in renal tissue between patients with IgAN and normal subjects using the Gene Expression Omnibus dataset and selected candidate mRNAs. mRNA expression in the urinary sediment was measured using quantitative real-time polymerase chain reaction. Associations between urinary mRNA levels and clinicopathological parameters were analyzed and the predictive value of mRNAs for disease progression was evaluated.

**Results:**

The urinary expression of *CCL2*, *CD14*, *DNMT1*, *FKBP5*, *Nephrin*, and *IL-6* was significantly upregulated in patients with IgAN compared with healthy controls. *C3*, *FLOT1*, and *Podocin* levels were significantly correlated with renal function, where *C3*, *FLOT1*, and *TfR* levels were significantly correlated with urinary protein excretion. During follow-up, 26 (13.0%) patients with IgAN experienced disease progression, defined as a greater than 50% reduction in the estimated glomerular filtration rate or progression to end-stage renal disease. Urinary mRNA levels of *FLOT1* (HR 3.706, 95% CI 1.373–10.005, *P* = 0.010) were independently associated with an increased risk of disease progression.

**Conclusions:**

Our results suggest that urinary sediment mRNAs are a useful biomarker in IgAN patients. Further studies with larger sample sizes and longer follow-up durations are required.

**Supplementary Information:**

The online version contains supplementary material available at 10.1186/s12882-024-03696-7.

## Introduction

IgA nephropathy (IgAN) is the most common primary glomerulonephritis and one of the leading causes of chronic kidney disease (CKD) worldwide [1, 2]. IgAN has a diverse geographical distribution and is frequently observed in East and Southeast Asia [1]. The symptoms and clinical course of IgAN vary, and more than one third of the patients progress to end-stage renal disease (ESRD) within 20 years after diagnostic biopsy [3]. Therefore, early diagnosis, individual risk stratification, and appropriate management are important for improving the prognosis of patients with IgAN. However, this remains a challenge because the pathogenesis of IgAN is complicated and not fully understood [4, 5].

The current gold standard for diagnosing and predicting disease progression in patients with IgAN is renal biopsy. However, owing to concerns about complications, such as bleeding and some clinical limitations, renal biopsy has not been performed routinely in patients suspected of having IgAN [6, 7]. In addition, even if a renal biopsy is performed, the pathologic findings may differ depending on the timing of the biopsy, and serial monitoring is often not feasible in several cases [8, 9]. Therefore, it is necessary to identify reliable biomarkers that are easily accessible, non-invasive, and provide predictive and prognostic information for IgAN.

Researchers have focused on urine as a potential platform for analyzing information on kidney diseases. Several studies have reported various urinary biomarkers that are associated with clinical and histological parameters and have the potential to predict the clinical outcomes of IgAN [10–12]. Recently, the exploration of urinary mRNAs has emerged as a promising field for identifying new biomarkers and understanding the pathogenesis of various kidney disorders [13–16]. However, only a few studies have investigated the clinical relevance of urinary mRNA levels in patients with IgAN [17, 18].

In this study, we selected IgAN disease-specific mRNA candidates by utilizing the public Gene Expression Omnibus (GEO) repository and a literature review, measured their urinary expression levels in patients with IgAN, and compared them with those in controls. Subsequently, we investigated the relationship between urinary mRNA levels and the clinicopathological parameters of patients with IgAN. The predictive value of each mRNA for CKD progression was also analyzed.

## Materials and methods

### Study population and design

This study enrolled 200 patients with biopsy-proven IgAN from two hospitals (Kyung Hee University Medical Center and Kyung Hee University Hospital at Gangdong) in Seoul, Korea between September 2010 and September 2019. We also enrolled patients with non-IgAN nephropathy as disease controls: six patients with lupus nephritis (LN), 16 with minimal change disease (MCD), 17 with crescentic glomerulonephritis, and nine patients with membranous nephropathy (MN). Patients on immunosuppressants prior to renal biopsy were not included in this study. Notably, 76 subjects without kidney disease were included as healthy controls. We compared the clinical characteristics, laboratory findings, and urinary mRNA expression levels between patients with IgAN and controls. Subsequently, we investigated the association between urinary mRNA expression levels and clinical and pathological parameters in patients with IgAN. To determine the prognostic value of each mRNA, patients with IgAN were divided into two groups according to CKD progression.

All the study procedures complied with the ethical guidelines of the Declaration of Helsinki and were approved by the Institutional Review Board of each hospital. The approval number from the Kyung Hee University Medical Center was 2020-11-029. Written informed consent was obtained from all participants.

### Clinical and pathological parameters

The baseline variables, including age, sex, body mass index, and prevalence of hypertension and diabetes mellitus, were recorded. Blood samples were collected for the measurement of serum albumin, IgA, and creatinine, and urine samples were collected to assess the amount of urinary protein excretion and the presence of hematuria at the time of renal biopsy. The amount of urinary protein excretion was calculated as the spot urine protein to creatinine ratio (PCR), and the estimated glomerular filtration rate (eGFR) was used to assess the renal function, calculated using the Chronic Kidney Disease Epidemiology Collaboration (CKD-EPI) equation [19]. The pathological findings of IgAN were described using the Oxford classification system [20].

### Treatment and clinical outcomes

Patients with IgAN were treated with angiotensin receptor blockers or angiotensin-converting enzyme inhibitors alone or in combination with immunosuppressants. All patients with IgAN regularly visited the outpatient clinic every 1–2 months for the assessment of renal function. The clinical outcome of this study was CKD progression, defined as a greater than 50% reduction in the eGFR from the value determined at the time of renal biopsy or progression to end-stage renal disease.

### Selection of IgAN disease-specific mRNA candidates

To select candidate mRNAs, we searched the keyword “IgA nephropathy” and “glomerulus” or “IgA nephropathy” and “tubulointerstitium” in the GEO database. Five data sets for glomerulus (GSE104948, GSE93798, GSE99339, GSE50469, and GSE37460) and five data sets for tubulointerstitium (GSE104954, GSE99340, GSE99325, GSE35488, and GSE35487) with the whole gene expression profiles of both IgAN patients and healthy controls were founded. The meta-analysis of these data sets was conducted using the GeneMeta R package, which follows the approach of Choi et al. [21] to identify significantly different genes between IgAN patients and healthy controls. Random-effects models were used for the meta-analysis. The false discovery rates (FDRs) were obtained from 1,000 permutations and the effective fold change (FC) of the meta-analysis was calculated as the average fold changes of the data sets weighted by the number of samples. Those with FC ≥ 2 or ≤ 0.5, and FDR < 0.001 were selected as the mRNA candidates in the meta-analysis data set. Among the 884 genes for the glomeruli and 67 genes for the tubulointerstitium with the lowest FDR, we selected eight genes (*C3*, *CD14*, *COL1A1*, *CX3CR1*, *DNMT1*, *FKBP5*, *FLOT1*, and *GDF15*) from the glomerulus and two genes (*CEBPD* and *PODXL*) from the tubulointerstitium. Additionally, we selected five genes (*CCL2*, *IL6*, *Nephrin*, *Podocin*, and *TfR*) as candidate markers based on previous studies and our pilot studies [17, 18, 22–25]. Finally, we measured the expression of 15 mRNA candidates in urine samples from the participants in the IgAN, disease control, and healthy control groups.

### Collection of urinary samples and measurement of urinary levels of mRNA

Urine samples were collected in sterile tubes on the day of renal biopsy (patients) or upon hospital visit (healthy controls). 50 mL of urine samples were centrifuged at 2,000 × g for 20 min at room temperature immediately after collection. The pellets were then transferred to RNAlater (Invitrogen, Carlsbad, CA, USA) and stored at -80 °C until use. All procedures were performed by an experienced technician immediately after urine sample collection. Total RNA was extracted from urinary pellets using a PureLink RNA Mini Kit (Invitrogen) according to the manufacturer’s recommendations. The amount of total RNA (ug) was measured using a NanoDrop^®^ ND-2000 UV spectrophotometer (Thermo Scientific, Waltham, MA). Reverse transcription was performed with the total RNA using M-MLV RT enzyme (200 U/µl; Mbiotech, Inc., Seoul, Korea), and the levels of gene expressions using each target primer and SYBR GreenMaster Mix (Applied Biosystems, Foster city, CA) were measured on ABI StepOne real-time polymerase chain reaction system (Applied Biosystems). Each mRNA level was normalized to glyceraldehyde 3-phosphate dehydrogenase (GAPDH), which was used as an endogenous control for the 2-DDCt method, and then log10-transformed to reduce deviation.

### Statistical analysis

Continuous variables are presented as the median (first quartile-third quartile) or means ± standard deviations as appropriate and categorical data are reported as the frequency and percentage. Continuous data were compared using the independent t-test or Mann-Whitney U test, as appropriate. Categorical data were compared using the Chi-square test. A Kruskal-Wallis test followed by a multiple comparison analysis was performed to analyze the differences in the clinical parameters among the glomerular disease groups. The Bonferroni test was used as appropriate for post-hoc analysis. Correlations between each urinary mRNA and the clinicopathological parameters were assessed using the Spearman’s rank correlation coefficient test. Univariate and multivariate Cox regression analyses were performed to identify the risk factors associated with CKD progression in patients with IgAN. Variables with a *P*-value < 0.10 in the univariate Cox regression analyses were selected for multivariate Cox regression analysis and results are presented as hazard ratios (HRs) ± 95% confidence intervals (CIs). All statistical analyses were conducted using SPSS software (version 19.0, SPSS Inc., Chicago, IL, USA). Statistical significance was set at *P* < 0.05.

## Results

### Baseline characteristics of study population

The baseline characteristics of the participants in the IgAN, disease control, and healthy control groups are presented in Table 1. The mean age of patients in the IgAN group was 42.4 years and 49.5% were male. The serum albumin levels were significantly higher in the IgAN group than in the control group. The renal function was significantly decreased in patients with crescentic glomerulonephritis compared to that in patients with IgAN and other disease controls. Patients with MN and MCD excreted significantly more urinary proteins than patients with IgAN. There were no significant differences in the prevalence of diabetes or hypertension between groups.

**Table 1:** Baseline characteristics of the study population according to the type of glomerular disease

	IgAN (*n* = 200)	LN (*n* = 6)	MCD (*n* = 16)	Crescentic GN (*n* = 17)	MN (*n* = 9)	Healthy control (*n* = 76)
**Age (years)**	42.38 ± 15.97^de^	39.16 ± 14.42 ^d^	42.68 ± 22.16 ^d^	66.59 ± 8.74 ^abc^	58.78 ± 10.92^a^	39.43 ± 15.21
**Male (n**,** %)**	99 (49.5%)	0 (0.0%)	9 (56.3%)	6 (35.3%)	5 (55.6%)	18 (23.7%)
**BMI (kg/m**^**2**^)	23.89 ± 3.43	22.4 ± 2.76	25.25 ± 3.77	23.52 ± 3.61	24.03 ± 1.63	-
**HTN (n**,** %)**	73 (36.5%)	2 (33.3%)	5 (31.3%)	6 (35.2%)	3 (33.3%)	-
**DM (n**,** %)**	12 (6.0%)	0 (0.0%)	1 (6.2%)	2 (11.8%)	1 (11.1%)	-
**Albumin (g/dL)**	3.90 ± 0.53^bcde^	2.87 ± 0.72^a^	2.51 ± 0.9^ade^	3.19 ± 0.48^ac^	3.20 ± 0.77^ac^	-
**IgA (mg/dL)**	305.11 ± 94.99	265.67 ± 117.46	273.06 ± 85.03	290.35 ± 101.09	210.33 ± 66.59	-
**Creatinine (mg/dL)**	1.32 ± 1.24 ^d^	0.94 ± 0.64 ^d^	1.20 ± 0.90 ^d^	4.18 ± 2.67 ^abce^	0.69 ± 0.21 ^d^	-
**eGFR (ml/min/1.73 m2)**	80.48 ± 37.23^d^	95.20 ± 49.81^d^	87.06 ± 43.45^d^	24.07 ± 31.38^abce^	99.24 ± 23.28 ^d^	-
**Urine PCR (g/gCr)**	1.64 ± 2.04 ^cef^	2.54 ± 2.26^cef^	9.84 ± 6.79 ^abdf^	2.26 ± 1.13^cef^	6.89 ± 4.71^abdf^	0.09 ± 0.06
**Urine RBC grade**						
< 5/HPF	45 (22.5%)	1 (16.7%)	10 (62.5%)	1 (5.9%)	3 (33.3%)	-
5–9/HPF	28 (14.0%)	1 (16.7%)	4 (25.0%)	2 (11.8%)	1 (11.1%)	-
10–29/HPF	47 (23.5%)	2 (33.3%)	1 (6.3%)	4 (23.5%)	2 (22.2%)	-
≥ 30/HPF	80 (40.0%)	2 (33.3%)	1 (6.3%)	10 (58.8%)	3 (33.3%)	-

IgAN, IgA nephropathy; LN, lupus nephritis; MCD, minimal change disease; Crescentic GN, crescentic glomerulonephritis; MN, membranous nephropathy; BMI, body mass index; HTN, hypertension; DM, diabetes mellitus; eGFR, estimated glomerular filtration rate; PCR, protein creatinine ratio; RBC, red blood cell; HPF, high-power field

a: *P* < 0.05, vs. IgAN; b: *P* < 0.05, vs. LN; c: *P* < 0.05, vs. MCD; d: *P* < 0.05, vs. Crescentic GN; e: *P* < 0.05, vs. MN; f: *P* < 0.05, vs. healthy control

### Levels of urinary mRNA candidates in different diagnostic groups

**Fig. 1:**
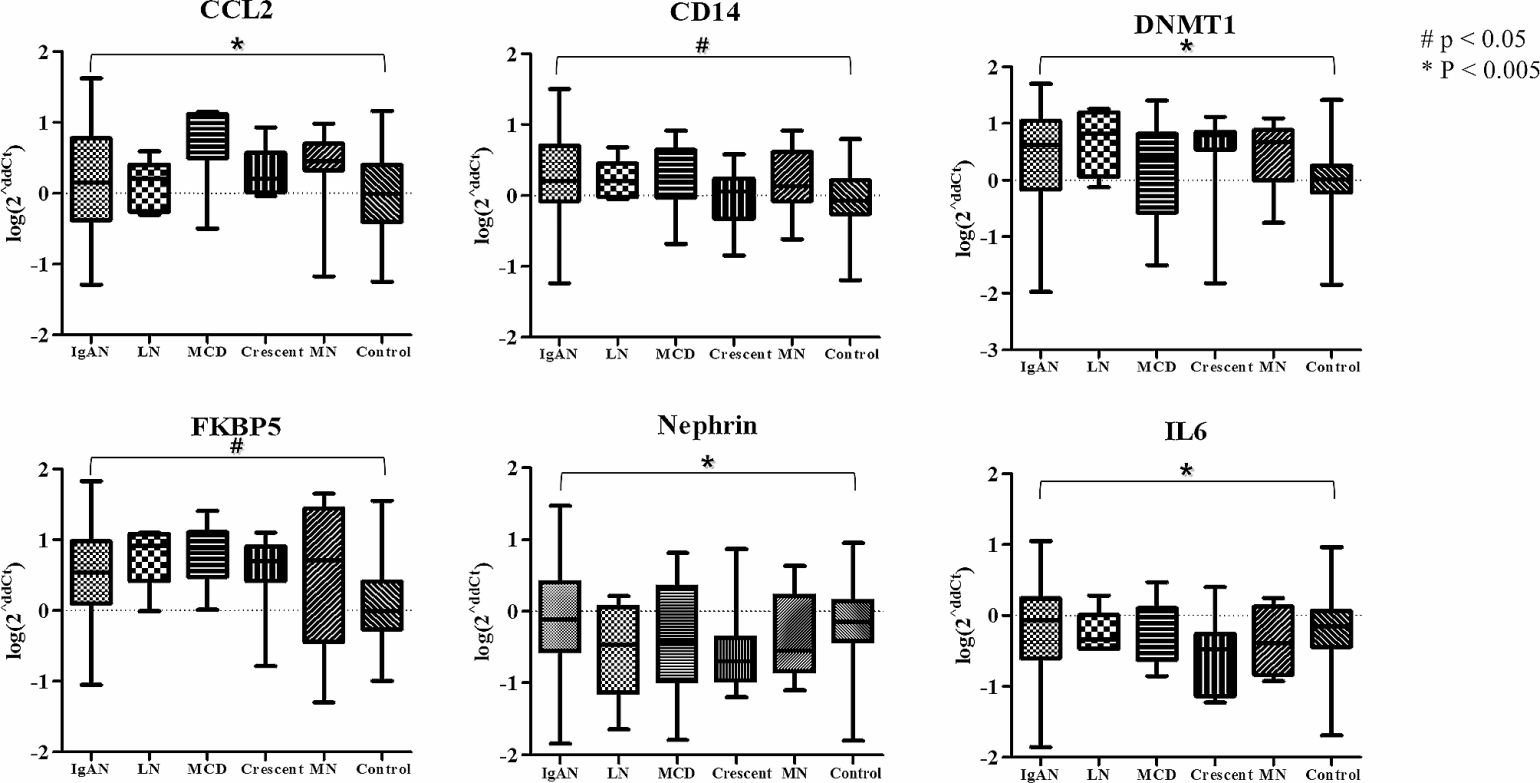
Urinary mRNAs significantly upregulated in IgAN patients compared to healthy controls

The levels of urinary candidate mRNAs were measured in urine samples from participants in the IgAN, disease control, and healthy control groups. The urinary mRNA expression levels of *CCL2*, *CD14*, *DNMT1*, *FKBP5*, *Nephrin*, and *IL-6* were significantly upregulated in patients with IgAN compared with healthy controls (Fig. 1). Although some urinary mRNA levels differed according to glomerular disease, we did not find significantly elevated IgAN mRNA expression (Supplementary Fig. 1).

### Association of urinary mRNA levels with clinical parameters in IgAN patients

**Fig. 2:**
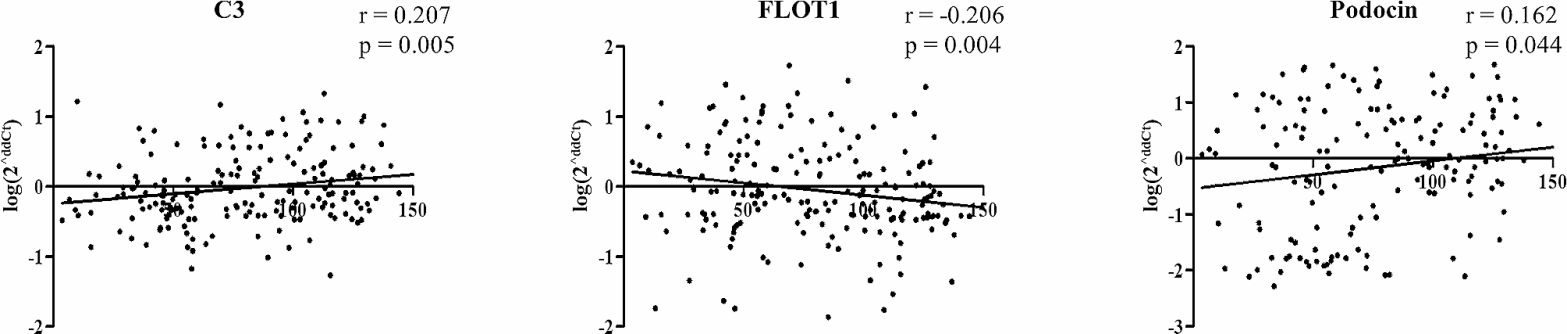
Urinary mRNAs significantly correlated with estimated glomerular filtration rate (mL/min/1.73 m^2^) in patients with IgA nephropathy

**Fig. 3:**
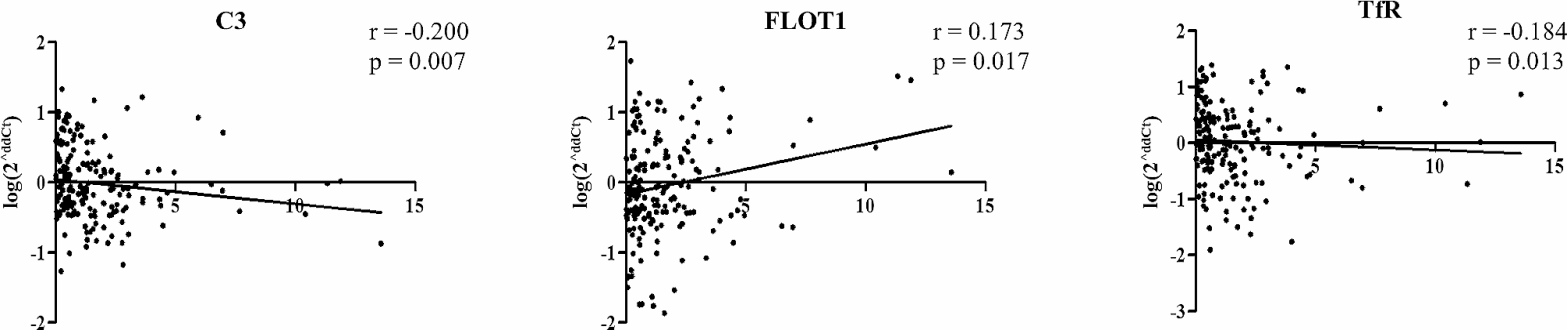
Urinary mRNAs significantly correlated with urinary protein excretion (urine protein-creatinine ratio, g/gCr) in patients with IgA nephropathy

The correlation between urinary mRNA expression levels and renal function in the 200 patients with IgAN were analyzed. As shown in Fig. 2, urinary *C3* and *Podocin* showed significant positive correlation with eGFR (*r* = 0.207, *P* = 0.005 and *r* = 0.162, *p* = 0.044, respectively). There was a significant negative correlation between urinary mRNA expression levels of *FLOT1* and eGFR (*r* = -0.206, *P* = 0.004). Other urinary mRNAs did not show a significant correlation with renal function (Supplementary Fig. 2). The relationship between urinary mRNA levels and urinary protein excretion was also analyzed. As shown in Fig. 3, urine PCR showed a negative correlation with urinary mRNA levels of *C3* and *TfR* (*r* = -0.200, *P* = 0.007 and *r* = -0.184, *P* = 0.013, respectively) and a positive correlation with urinary mRNA levels of *FLOT1* (*r* = 0.173, *P* = 0.017). Other urinary mRNAs did not show a significant correlation with urinary protein excretion (Supplementary Fig. 3). All the coefficient values and distribution patterns suggest that the correlation power is not strong. Additionally, we investigated the association between the quantity of urinary red blood cells and mRNA levels. However, our findings did not reveal any significant discrepancies. (Supplementary Fig. 4).

### Association of urinary mRNA levels with pathological findings in IgAN patients

**Fig. 4:**
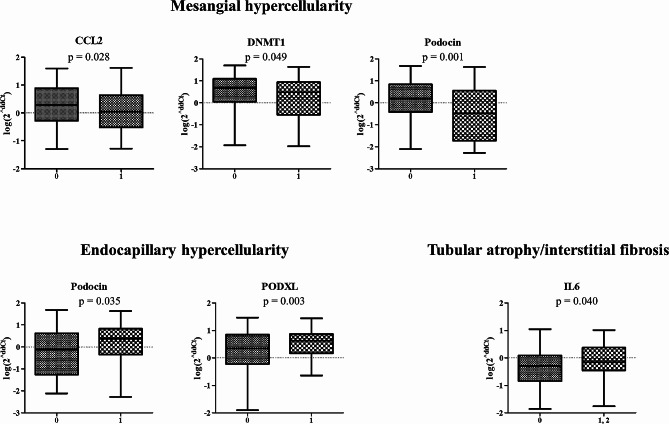
Expression of urinary sediment mRNA according to pathologic findings based on the Oxford classification

We also analyzed the association between urinary mRNA expression levels and pathological findings based on the Oxford classification (Fig. 4). The urinary mRNA levels of *CCL2*, *DNMT1* and *Podocin* were significantly decreased in patients with mesangial hypercellularity (*P* = 0.028, *P* = 0.049, and *P* = 0.001, respectively). In patients with endocapillary hypercellularity, urinary mRNA levels of *Podocin* and *PODXL* were significantly elevated (*P* = 0.035 and *P* = 0.003, respectively). Urinary mRNA levels of *IL-6* were significantly elevated in patients with tubular atrophy/interstitial fibrosis (*P* = 0.040). No mRNA showed a significant relationship with segmental glomerulosclerosis or cellular or fibrocellular crescents.

### Association of urinary mRNA levels and CKD progression in IgAN patients

During a mean follow-up period of 35 months, 26 patients (13.0%) experienced CKD progression. CKD progression was defined as a greater than 50% reduction in eGFR or progression to end-stage renal disease. Patients with IgAN were divided into two groups according to CKD progression; the characteristics of the two groups are shown in Table 2. Patients in the CKD progression group were significantly older and had a higher proportion of male sex than those in the non-CKD progression group. Patients in the CKD progression group had a higher prevalence of hypertension (*P* < 0.001), lower serum albumin levels and eGFR (*P* < 0.001 and *P* < 0.001, respectively), and higher urinary PCR levels (*P* < 0.001). Patients in the CKD progression group had a higher use of immunosuppressants after renal biopsy than patients in the non-CKD progression group. The types of immunosuppressant used were not significantly different between the two groups (Supplementary Table 1). There was no significant difference in the use of angiotensin II receptor blockers or angiotensin-converting enzyme inhibitors between the two groups. Mesangial hypercellularity, tubular atrophy/interstitial fibrosis, and cellular or fibrocellular crescents were more frequently observed in the CKD progression group (*P* = 0.001, *P* < 0.001, and *P* = 0.006, respectively).

**Table 2:** Clinical characteristics of the IgAN patients according to disease progression

	Progression (*n* = 26)	Non-progression (*n* = 174)	*P*
**Age (years)**	51.31 ± 19.08	41.05 ± 15.06	0.014
**Male (n**,** %)**	18 (69.2%)	81 (46.6%)	0.025
**BMI (kg/m**^**2**^)	24.51 ± 3.17	23.81 ± 3.46	0.347
**HTN (n**,** %)**	20 (76.9%)	53 (30.5%)	< 0.001
**DM (n**,** %)**	1 (3.8%)	11 (6.3%)	0.502
**Albumin (g/dL)**	3.52 ± 0.63	3.96 ± 0.50	< 0.001
**IgA (mg/dL)**	282.69 ± 87.78	308.48 ± 95.82	0.198
**eGFR (ml/min/1.73 m**^**2**^)	33.25 ± 15.64	87.54 ± 34.26	< 0.001
**Urine PCR (g/gCr)**	3.72 ± 2.97	1.33 ± 1.67	< 0.001
**Use of ARB or ACEi before renal biopsy (n**,** %)**	9 (34.6%)	49 (28.2%)	0.499
**Use of ARB or ACEi after renal biopsy (n**,** %)**	22 (84.6%)	123 (70.7%)	0.103
**Use of immunosuppressant after renal biopsy (n**,** %)**	23 (88.5%)	105 (60.3%)	0.003
**Urine RBC grade (n**,** %)**			0.054
< 5/HPF	7 (25.9%)	38 (21.8%)	
5–9/HPF	4 (14.8%)	24 (13.8%)	
10–29/HPF	11 (40.7%)	36 (20.7%)	
≥ 30/HPF	5 (18.5%)	76 (37.8%)	
**Oxford classification M**			0.001
0	6 (23.1%)	103 (59.2%)	
1	20 (76.9%)	70 (40.8%)	
**Oxford classification E**			0.238
0	17 (65.4%)	129 (74.1%)	
1	9 (34.6%)	45 (25.9%)	
**Oxford classification S**			0.367
0	17 (65.4%)	123 (70.7%)	
1	9 (34.6%)	51 (29.3%)	
**Oxford classification T**			< 0.001
0	14 (53.8%)	158 (90.8%)	
1	12 (46.2%)	16 (9.2%)	
**Oxford classification C**			0.006
0	14 (53.8%)	139 (79.9%)	
1	12 (46.2%)	35 (20.1%)	

BMI, body mass index; HTN, hypertension; DM, diabetes mellitus; eGFR, estimated glomerular filtration rate; PCR, protein creatinine ratio; ARB, angiotensin II receptor blockers; ACEi, angiotensin II-converting enzyme inhibitors; RBC, red blood cell; HPF, high-power field

Univariate and multivariate Cox regression analyses were conducted to identify the risk factors associated with CKD progression (Table 3). In the univariate Cox regression analysis, age, male sex, hypertension, serum albumin levels, eGFR, urine PCR, immunosuppressant use, mesangial hypercellularity, tubular atrophy/interstitial fibrosis, cellular or fibrocellular crescents, and urinary mRNA levels of *FLOT1*, *Nephrin*, and *TfR* showed a significant association with CKD progression in patients with IgAN. Multivariate analysis was conducted by adjusting for variables with a *P*-value of less than 0.10 in the univariate analysis. These variables included age, male sex, hypertension, serum albumin levels, eGFR, urine PCR, immunosuppressants use, mesangial hypercellularity, endocapillary hypercellularity, tubular atrophy/interstitial fibrosis, cellular or fibrocellular crescents, and urinary mRNA levels of FLOT1, Nephrin, and TfR. In multivariate analysis, eGFR (HR 0.937, 95% CI 0.904–0.973, *P* = 0.001), urine PCR (HR 1.357, 95% CI 1.019–1.808, *P* = 0.037), and urinary mRNA levels of *FLOT1* (HR 3.706, 95% CI 1.373–10.005, *P* = 0.010) were independently associated with CKD progression in patients with IgAN.

**Table 3:** Prediction of disease progression in univariate and multivariate Cox regression analyses

	Univariate		Multivariate^*^	
	HR (95% CI)	*P*	HR (95% CI)	*P*
**Age (years)**	1.044 (1.016–1.074)	0.002	0.962 (0.924–1.001)	0.058
**Male**	2.324 (1.009–5.353)	0.048	2.743 (0.957–7.861)	0.060
**BMI (kg/m**^**2**^)	1.051 (0.934–1.183)	0.404		
**HTN**	5.621 (2.254–14.014)	< 0.001	1.004 (0.196–5.153)	0.996
**DM**	0.635 (0.086–4.701)	0.656		
**Albumin (g/dL)**	0.373 (0.234–0.581)	< 0.001	0.450 (0.123–1.651)	0.229
**IgA (mg/dL)**	0.998 (0.994–1.002)	0.313		
**eGFR (ml/min/1.73 m**^**2**^)	0.936 (0.915–0.958)	< 0.001	0.937 (0.904–0.973)	0.001
**Urine PCR (g/gCr)**	1.384 (1.246–1.537)	< 0.001	1.357 (1.019–1.808)	0.037
**Use of ARB or ACEi before renal biopsy**	1.443 (0.642–3.244)	0.376		
**Use of ARB or ACEi after renal biopsy (n**,** %)**	1.815 (0.623–5.288)	0.275		
**Use of immunosuppressants after renal biopsy**	5.715 (1.670-19.552)	0.005	0.724 (0.104–4.978)	0.745
**Urine RBC grade)**				
< 5/HPF	1			
5–9/HPF	0.667 (0.195–2.284)	0.519		
10–29/HPF	1.144 (0.442–2.957)	0.782		
≥ 30/HPF	0.303 (0.089–1.035)	0.157		
**Oxford classification M**				
0	1			
1	3.216 (1.284–8.055)	0.013	2.318 (0.738–7.449)	0.394
**Oxford classification E**				
0	1			
1	1.991 (0.880–4.506)	0.098	4.508 (0.895–22.702)	0.068
**Oxford classification S**				
0	1			
1	1.213 (0.537–2.739)	0.643		
**Oxford classification T**				
0	1			
1, 2	4.978 (2.297–10.788)	< 0.001	1.837 (0.453–7.449)	0.394
**Oxford classification C**				
0	1			
1, 2	3.009 (1.390–6.515)	0.005	0.709 (0.193–2.614)	0.606
**C3**	0.618 (0.259–1.477)	0.279		
**CCL2**	1.179 (0.661–2.091)	0.582		
**CD14**	1.048 (0.533–2.064)	0.891		
**CEBPD**	1.687 (0.889–3.202)	0.110		
**COL1A1**	1.103 (0.652–1.868)	0.714		
**CX3CR1**	1.409 (0.766–2.591)	0.270		
**DNMT1**	0.975 (0.644–1.476)	0.904		
**FKBP5**	0.910 (0.484–1.712)	0.770		
**FLOT1**	2.431 (1.436–4.113)	0.001	3.706 (1.373–10.005)	0.010
**GDF15**	0.645 (0.350–1.189)	0.159		
**IL6**	1.370 (0.749–2.507)	0.307		
**Nephrin**	2.023 (1.145–3.572)	0.015	1.902 (0.787–4.124)	0.103
**Podocin**	1.076 (0.729–1.587)	0.712		
**PODXL**	0.940 (0.490–1.804)	0.853		
**TfR**	0.546 (0.307–0.970)	0.039	0.513 (0.245–1.076)	0.077

BMI, body mass index; HTN, hypertension; DM, diabetes mellitus; eGFR, estimated glomerular filtration rate; PCR, protein creatinine ratio; ARB, angiotensin II receptor blockers; ACEi, angiotensin II-converting enzyme inhibitors; RBC, red blood cell; HPF, high-power field

^*^ Multivariate analysis was performed by adjusting for variables with a *P*-value of less than 0.10 in the univariate analysis

## Discussion

In this study, we selected 15 mRNAs as candidate biomarkers for IgAN using public GEO data-sets and a literature review. We measured the urinary expression levels of these mRNAs and investigated their clinical significance in patients with IgAN to identify reliable noninvasive biomarkers which could predict the disease course. Among the 15 mRNAs, the urinary expression of six mRNAs (*CCL2*, *CD14*, *DNMT1*, *FKBP5*, *Nephrin*, and *IL-6*) was significantly higher in patients with IgAN than in healthy controls. Some RNAs were significantly correlated with renal function and urinary protein excretion. There were differences in the expression of some RNAs according to pathological severity.

Advances in omics technologies have helped us understand the biological pathways of diseases and facilitate access to potential treatment targets and biomarkers. Identifying differentially expressed genes between diseased and control samples using array-based gene expression analysis has emerged as a useful tool for identifying novel biomarkers of several disease conditions [26]. Gene expression analyses in kidney tissues have helped to understand the pathogenesis of various kidney diseases [27]. Recent studies have reported that not only kidney tissue but also urine is a potential source for gene expression analyses [9].

Urine examination plays an important role in the diagnosis of kidney disease and the prognosis [28]. Urine is easy to obtain and available in large quantities using a non-invasive collection method. Since changes and ongoing injury of the kidney are well reflected in urine, urine substances, such as proteins, blood cells, and casts provide valuable clues for understanding kidney disorders [29]. The urine substances can be indicative of both filtered and locally produced proteins within the renal tissue. Consequently, they provide insight into the systemic condition, glomerular permeability, renal cellular activity, and renal pathologies. Several peptides present in urine have been suggested as disease-specific biomarkers of various kidney diseases [30]. Recently, quantification of the mRNAs expression of target genes in urine has been suggested as a potential non-invasive diagnostic and prognostic marker for multiple types of kidney disorder [13, 15, 16]. Our study group previously reported the clinical relevance of urinary mRNAs in various kidney conditions including diabetic kidney disease and rejection of transplanted kidney [14, 31, 32].

Considering that genetic factors influence the development of IgAN [33, 34], gene expression studies can be helpful in understanding the disease pathogenesis and identifying the novel biomarkers of IgAN. Few studies have investigated urinary mRNA as a non-invasive biomarker of IgAN. Fukuda et al. [17] showed that urinary podocyte mRNA levels correlated with segmental glomerulosclerosis and acute extracapillary proliferative lesions at the time of renal biopsy and decreased after treatment. The authors suggested that podocyte mRNAs serve as a useful marker for detecting and monitoring disease activity during treatment in patients with IgAN. Other researchers observed that the mRNA expression of *C-C motif chemokine ligand 2* (*CCL2*) was highly induced in the urine of patients with IgAN compared to controls. The mRNA level of *CCL2* in urine is closely correlated with the severity of pathologic changes documented by renal biopsy and with the deterioration of renal function [18].

Consistent with previous studies, our findings suggest that urinary mRNAs may be novel and promising biomarkers for IgAN. The urinary expression of *C3*, *FLOT1*, and *Podocin* was significantly correlated with renal function, where urinary expression of *C3*, *FLOT1*, and *TfR* was significantly correlation with urinary protein excretion. The urinary expression levels of *CCL2*, *DNMT1* and *Podocin* are affected by the severity of mesangial hypercellularity. *Podocin* and *PODXL* showed higher expression levels in patients with endocapillary hypercellularity, and *IL-6* expression was significantly increased in patients with tubular atrophy/interstitial fibrosis. Urinary mRNA expression did not show significant sex differences in our study population. Subgroup analyses stratified by sex showed results consistent with those observed in the overall study population.

In our study, the mRNA level of *FLOT1* in the urine showed a significant correlation with renal function and proteinuria and has a predictive value for CKD progression. FLOT1 is located at 6p21.3 and encodes flotillin-1. It has been reported that flotillin-1 was involved in diverse biological processes including cell proliferation, cell adhesion, molecular signal transduction, T-lymphocyte activation, endocytosis, and the regulation of axons [35, 36]. Recent studies have shown the overexpression of flotillin-1 in various types of malignancies and have demonstrated that flotillin is associated with tumor development and metastasis [37, 38]. However, no study has reported an association between flotillin-1 and IgAN. A Receiver Operating Characteristic (ROC) curve analysis was conducted to assess the clinical utility of incorporating FLOT1 into the International IgA Nephropathy Risk Prediction Tool. However, no significant differences were identified (Supplementary Fig. 5). Further research is required to elucidate the underlying mechanisms of our findings and to determine the clinical utility of FLOT1 in IgAN.

Our study has some potential limitations. First, we selected mRNA candidates using GEO datasets based on comparisons of IgAN and healthy controls; therefore, the mRNAs might reflect not only IgAN, but also other kidney disorders. Although the expression of some mRNAs was significantly different between the IgAN and healthy control groups in our study, we could not find significantly elevated mRNA expression in IgAN only. Second, because the urinary expression levels of mRNAs were measured only once, we could not observe changes in the expression of mRNAs according to treatment and were not able to investigate the monitoring function of mRNAs. In our next study, we plan to eliminate these drawbacks using prospective cohort with multiple data collections. Finally, due to the relatively small number of study subjects, we were unable to identify the clinical significance of mRNA in disease controls. In future research, we intend to address this limitation by enrolling a larger number of subjects with various glomerular diseases.

In conclusion, we selected candidate IgAN mRNAs and measured their urine expression levels, and investigated their clinical significance. Our findings suggest that urinary mRNA expression signatures may serve as useful biomarkers of IgAN. The identification of mRNA in urine may assist in differentiating between proteinuria resulting from active inflammation and chronic changes. This distinction is crucial for clinical applications, as it enables more precise monitoring of disease activity and progression. By identifying the underlying cause of proteinuria, clinicians can more effectively tailor treatment strategies. The ability to non-invasively assess active inflammation versus chronic damage through urinary mRNA analysis provides a significant advantage in the management of IgAN. Further studies with larger sample sizes and prospective designs are needed to validate and ascertain the underlying mechanisms.

## Electronic supplementary material

Below is the link to the electronic supplementary material.

Supplementary Material 1

Supplementary Material 2

Supplementary Material 3

Supplementary Material 4

Supplementary Material 5

Supplementary Material 6

## Data Availability

The datasets generated and analysed in the current study are available from the corresponding author upon reasonable request.
