# Supplementary material for: Urinary sediment mRNA as a potent biomarker of IgA nephropathy
Source: BMC Nephrol. 2024 Nov 8;25:401. doi: 10.1186/s12882-024-03696-7 (PMC11549797; doi:10.1186/s12882-024-03696-7)
Supplement: Supplementary file 1 — Supplementary Material 1 [file 12882_2024_3696_MOESM1_ESM.docx]

**Urinary sediment mRNA as a potent biomarker of IgA nephropathy**

**Supplementary Table 1**

**Supplementary Figure legends**

**Supplementary Table 1. Types of immunosuppressant use according to the disease progression**

|  |  | **Progression**  **(n = 26)** | **Non-progression**  **(n = 174)** | ***P*** |
| --- | --- | --- | --- | --- |
| **Types of immunosuppressant (n, %)** |  |  |  | 0.985 |
| Steroid only |  | 16 (69.6%) | 74 (70.5%) |  |
| Steroid and others* |  | 5 (21.7%) | 23 (21.9%) |  |
| Others* |  | 2 (8.7%) | 8 (7.6%) |  |

* Others include mycophenolate mofetil, cyclosporine, cyclophosphamide, and azathioprine.

**Supplementary Figure legends**

**Supplementary Figure 1. Expression of urinary sediment mRNA according to the study group.**

**Supplementary Figure 2. Relationships between the expression of urinary mRNA and estimated glomerular filtration rate (mL/min/1.73 m^2^) in patients with IgA nephropathy.**

**Supplementary Figure 3. Relationships between the expression of urinary mRNA and urinary protein excretion (urine protein-creatinine ratio, g/gCr) in patients with IgA nephropathy.**

**Supplementary Figure 4. Expression of urinary sediment mRNA according to the amount of urinary red blood cells.**

**Supplementary Figure 5. Receiver Operating Characteristic (ROC) curves for disease progression in patients with IgA nephropathy.**
