# Supplementary figures and images for: Urinary sediment mRNA as a potent biomarker of IgA nephropathy
Source: BMC Nephrol. 2024 Nov 8;25:401. doi: 10.1186/s12882-024-03696-7 (PMC11549797; doi:10.1186/s12882-024-03696-7)

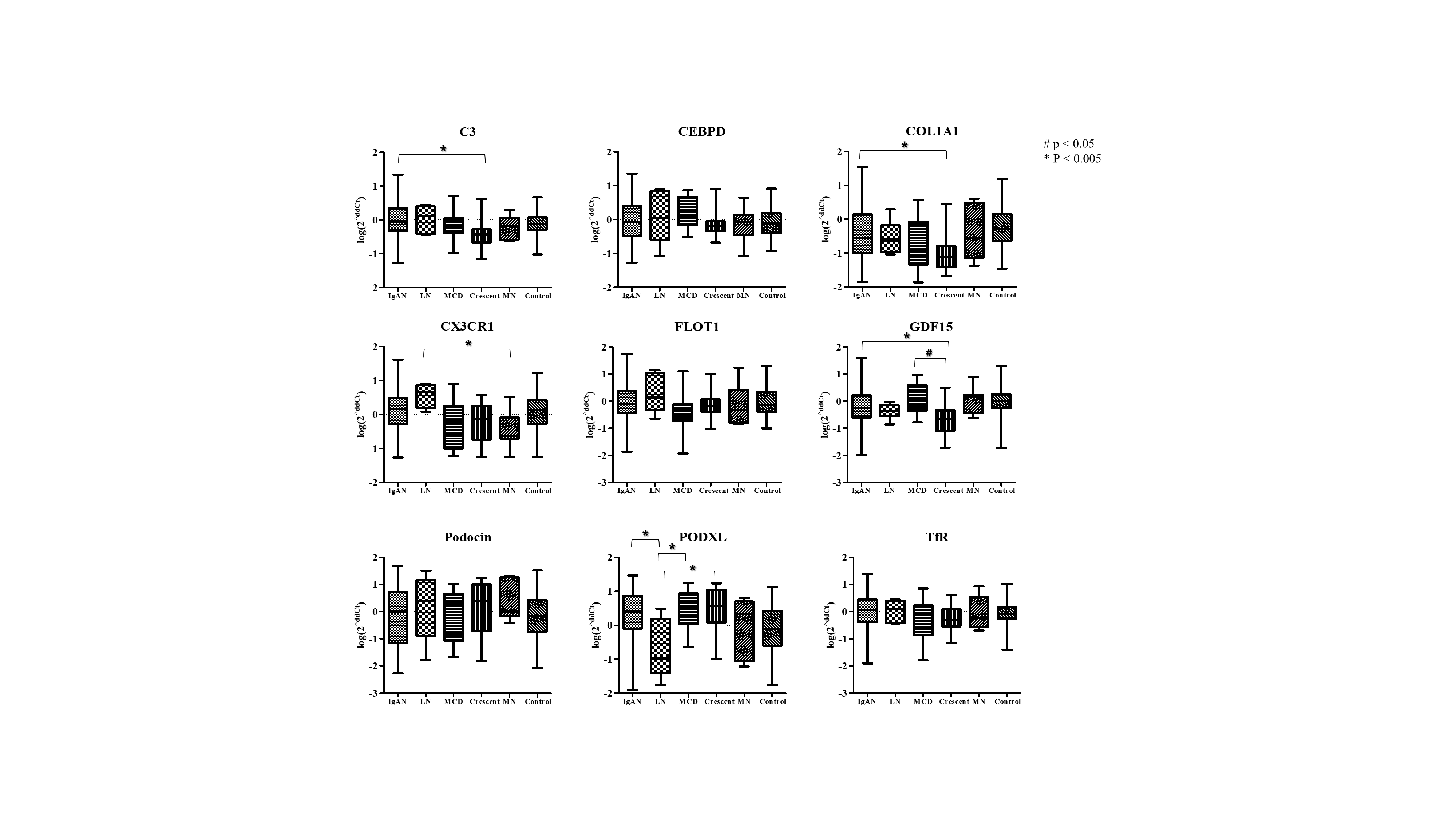

Supplement: Supplementary file 2 — Supplementary Material 2 [file 12882_2024_3696_MOESM2_ESM.png]

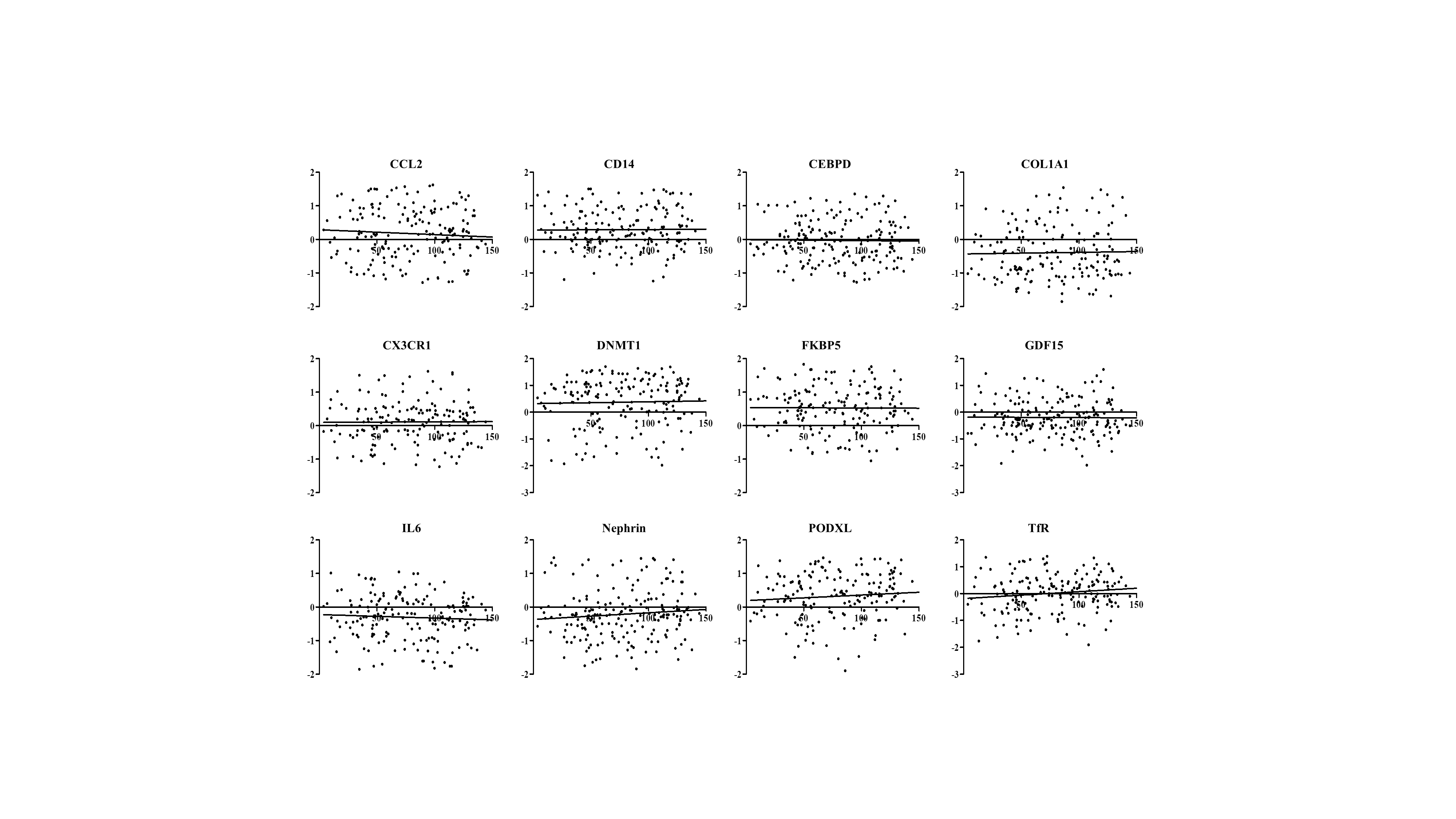

Supplement: Supplementary file 3 — Supplementary Material 3 [file 12882_2024_3696_MOESM3_ESM.png]

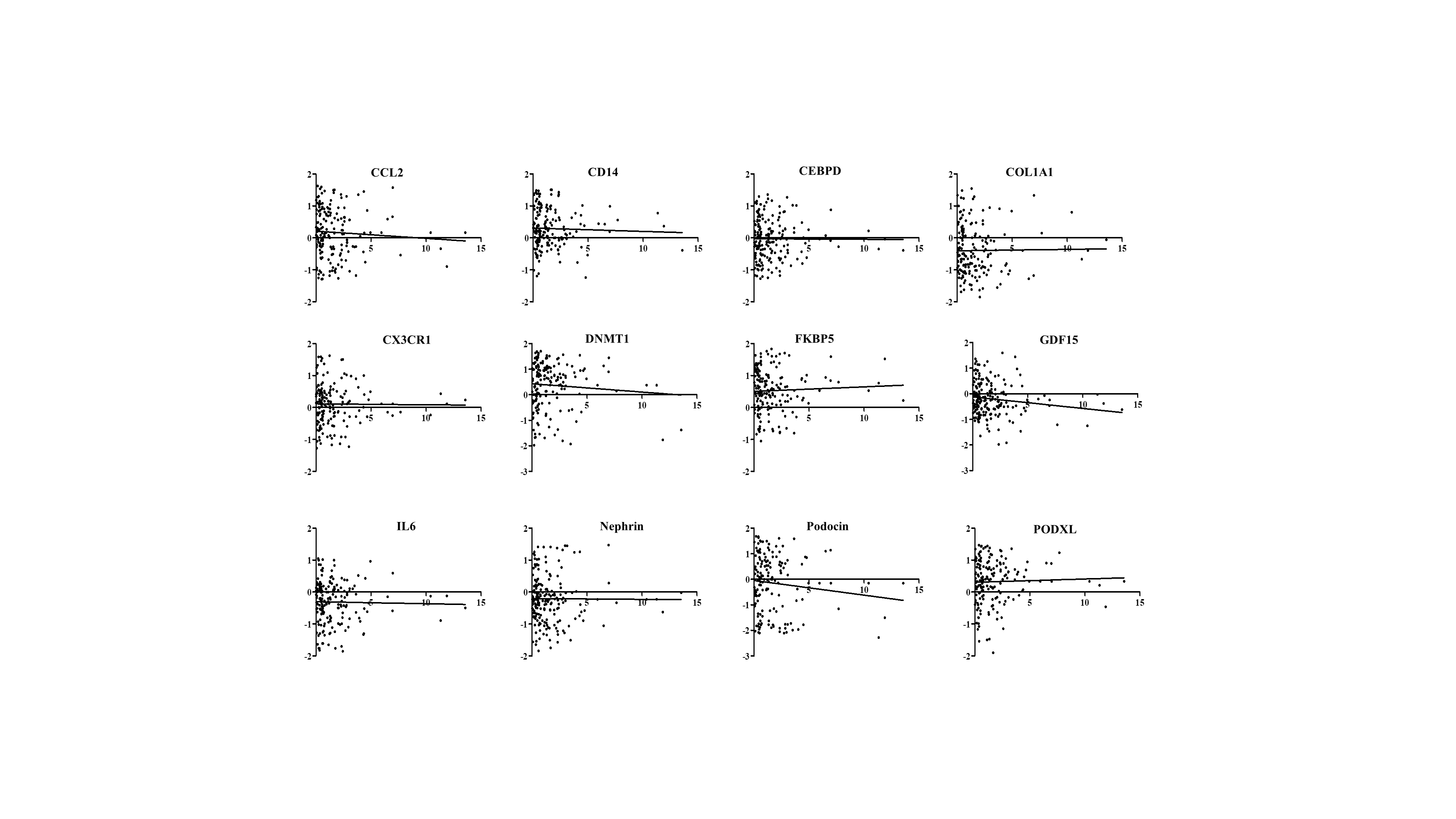

Supplement: Supplementary file 4 — Supplementary Material 4 [file 12882_2024_3696_MOESM4_ESM.png]

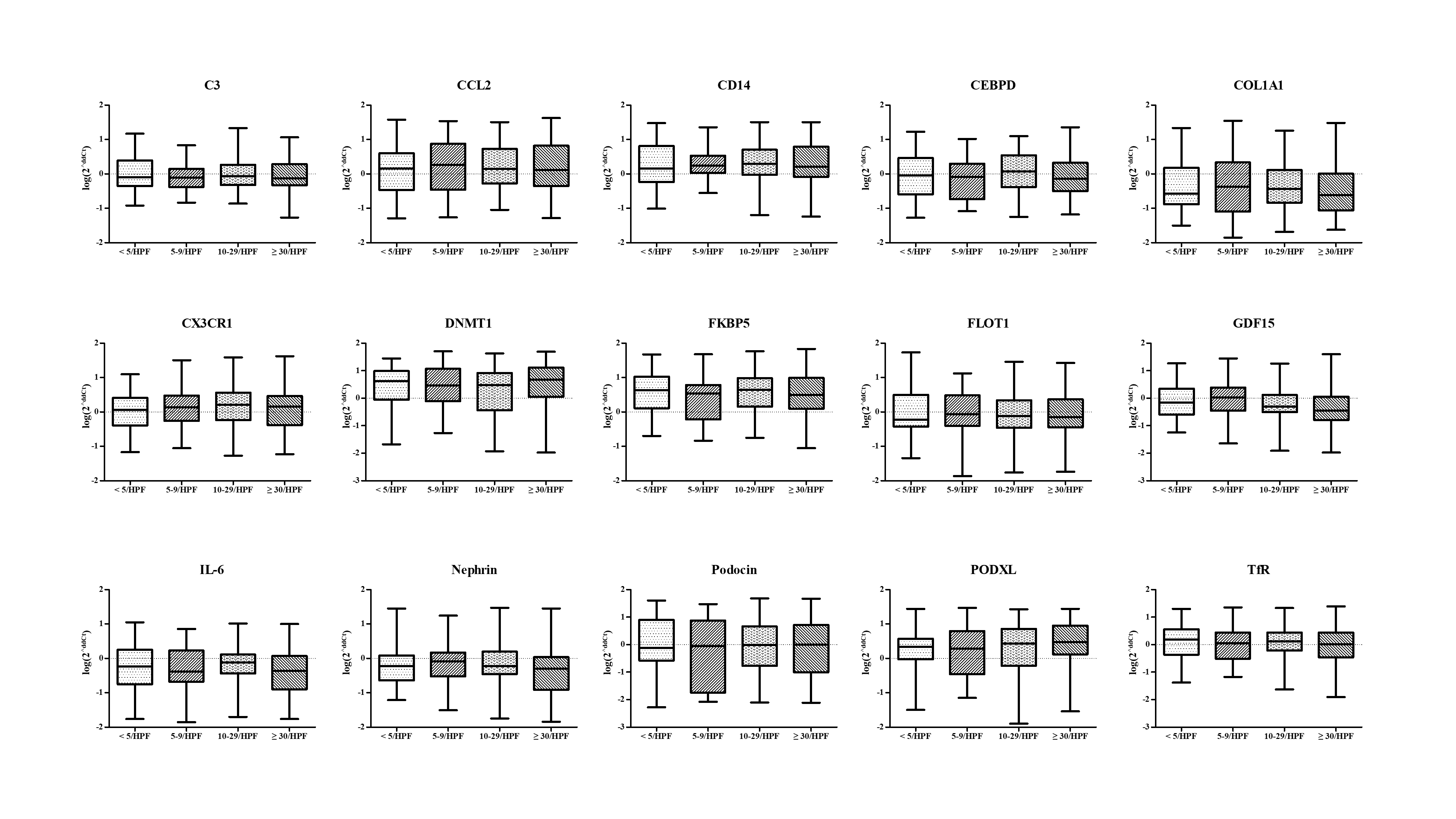

Supplement: Supplementary file 5 — Supplementary Material 5 [file 12882_2024_3696_MOESM5_ESM.png]

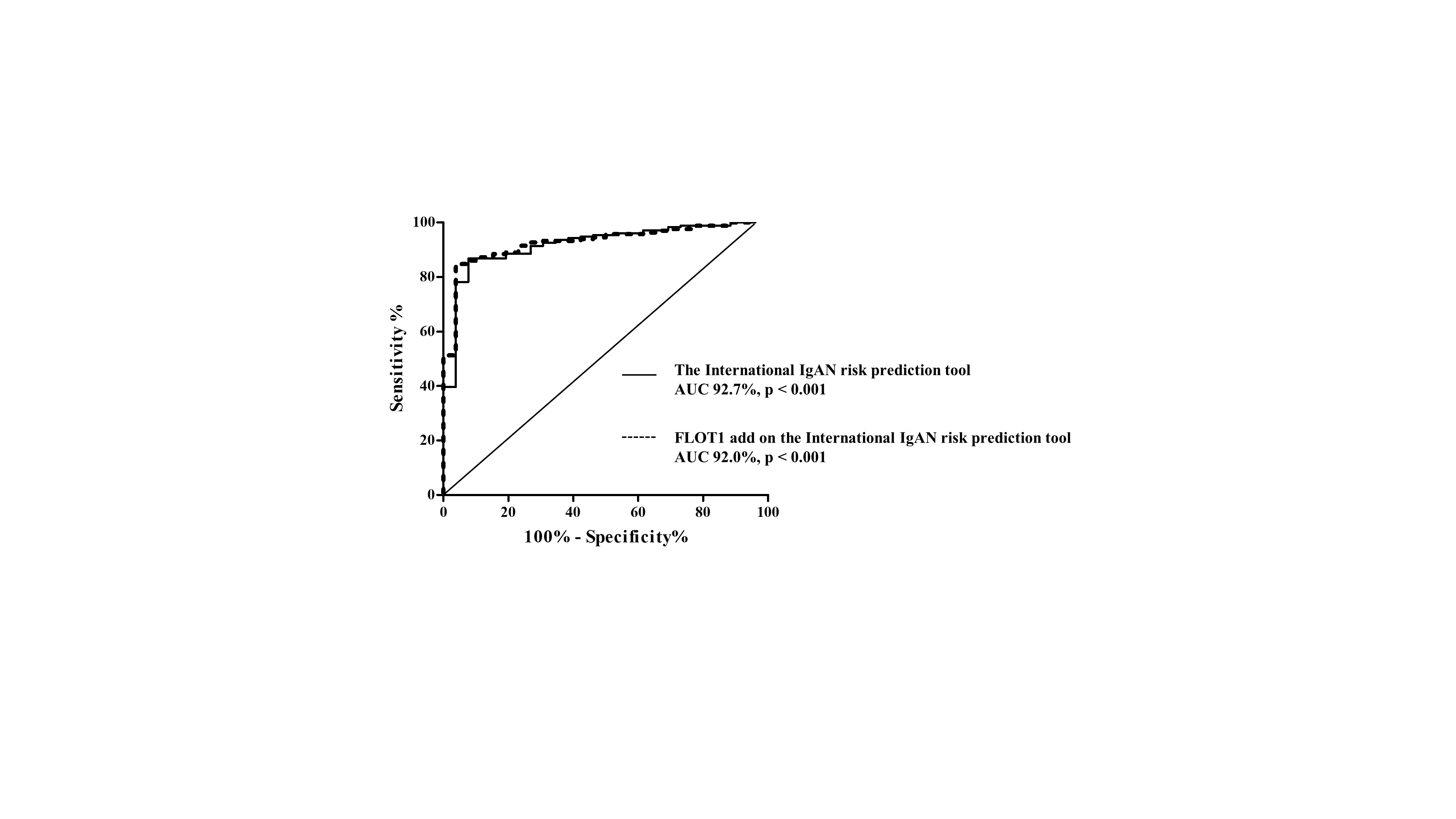

Supplement: Supplementary file 6 — Supplementary Material 6 [file 12882_2024_3696_MOESM6_ESM.png]
